# Pregnancy and lactation exposure to bisphenol S induces ferroptosis via disrupted hepatic lipid metabolism in offspring mice

**DOI:** 10.3389/ftox.2026.1753029

**Published:** 2026-06-05

**Authors:** Mengfen Pan, Xinxin Guo, Caiyun Wei, Nuo Xu, Yan Su, Zhensong Ma, Huaicai Zeng

**Affiliations:** 1 Guangxi Key Laboratory of Environmental Exposomics and Entire Lifecycle Health, Guilin Medical University, Guilin, China; 2 Department of Environmental and Occupational Health, School of Public Health, Guilin Medical University, Guilin, China

**Keywords:** bisphenol S, ferroptosis, liver damage, mitochondrial damage, oxidative stress

## Abstract

**Introduction:**

Bisphenol S (BPS), a widely used environmental endocrine disruptor, induces multigenerational liver injury, though its underlying mechanisms remain unclear.

**Methods:**

In this study, pregnant mice were housed separately and randomly divided into control and BPS (0.2, 2, and 20 mg/L) group through drinking water exposure. We observed the pathological changes in the livers of the offspring mice and the indicators of liver lipid metabolism disorder. At the same time, we also measured the levels of liver peroxidation and ferroptosis. Additionally, we established a co-exposure situation of in vitro BPS and ferroptosis inhibitors (Fer-1), analyzed the expression of ferroptosis-related genes to evaluate the hepatotoxicity caused by BPS by disrupting liver lipid metabolism and inducing iron depletion.

**Results:**

In this study, we observed that BPS exposure induced liver injury and significantly impaired hepatic function in offspring, as indicated by elevated serum ALT and AST levels. Perinatal BPS exposure altered the expression of genes involved in fatty acid β- oxidation, synthesis, and absorption, and concurrently induced dyslipidemia characterized by elevated triglyceride, total cholesterol, and LDL-cholesterol levels and reduced HDL-cholesterol. We further established that BPS promotes ferroptosis in offspring mice, as evidenced by iron accumulation, mitochondrial damage, and oxidative damage. Mechanistically, BPS increased the expression of genes related to lipid peroxidation while suppressing those involved in the antioxidant system. Notably, Fer-1 cotreatment markedly alleviated both the lipid metabolism disorders and ferroptosis triggered by BPS.

**Discussion:**

this study provides evidence that perinatal BPS exposure leads to hepatic lipid metabolism dysfunction and ferroptosis activation in offspring mice, highlighting BPS as a potential hepatotoxicant.

## 1 Introduction

Bisphenols, a category of synthetic organic compounds, are extensively utilized in the production of various industrial and consumer products, including polycarbonate plastics, epoxy resins, thermal papers, and flame retardants (Heinsberg et al., 2020; Vandenberg et al., 2009). Among them, bisphenol A (BPA) has attracted global attention due to its potential neurotoxicity, reproductive toxicity, carcinogenicity, and other health risks (Gebru and Pang, 2023; Rezg et al., 2022; Wang et al., 2023). Following the widespread prohibition of BPA in numerous countries, structural analogs such as bisphenol S (BPS) have been increasingly utilized as substitute materials. It is noteworthy that this compound has been frequently detected in human body fluids (urine, serum, human breast milk, and fetal developmental environments (Hao et al., 2021; Lee et al., 2022; Tuzimski et al., 2018). Multiple studies have indicated that exposure to bisphenol compounds during pregnancy and lactation not only exerts toxic effects on the parental generation but also adversely impacts offspring development (Dias et al., 2025). Recent toxicological evidence demonstrates a significant dose-response relationship between BPS exposure and the incidence of metabolic disorders (Blaauwendraad et al., 2021). Its perinatal exposure to BPS may increase the risk of metabolic syndromes in offspring by disrupting metabolic homeostasis and developmental processes (Ahn et al., 2020; Molangiri et al., 2022). However, their potential toxicological effects remain a contentious issue in ongoing scientific discourse.

Epidemiological studies have reported an association between bisphenol exposure and non-alcoholic fatty liver disease and chronic exposure to BPS induced significant hepatic lipid accumulation and dyslipidemia in mice (Li S. et al., 2025). BPS exposure also has been found to significantly alter lipid metabolic pathways in the liver, resulting in increased lipid accumulation and lipid peroxidation (Li Z. et al., 2023). This disrupted lipid metabolism not only directly impairs hepatocyte health but may also exacerbate oxidative stress, creating a vicious cycle that ultimately leads to hepatocyte injury and dysfunction (Xie et al., 2025). BPS induced lipid metabolism disorders and hepatotoxicity are closely related to the aberrant activation of multiple intracellular signaling pathways. For example, BPS impair lipid metabolism and induce lipid accumulation by inhibiting the expression of PPARα (peroxisome proliferator-activated receptor α), thereby affecting the expression of downstream target genes such as CPT1b (carnitine palmitoyltransferase 1b) (Lin et al., 2025). Additionally, BPS may exacerbate lipid accumulation by inhibiting PPARα expression and downregulating lipid metabolism-related genes. SREBP1C is an important transcription factor for lipid synthesis, and its overexpression promotes triglyceride (TG) accumulation in hepatocytes (Peña de la Sancha et al., 2025). BPS exposure significantly upregulates SREBP1C and FASN expression, thereby promoting lipid synthesis and leading to lipid accumulation.

Lipid accumulation drives the occurrence of ferroptosis by promoting lipid peroxidation and iron ion accumulation (Peng et al., 2025). BPS may exacerbate liver injury by disrupting intracellular iron metabolism and increasing intracellular iron accumulation and system Xc^−^ plays a crucial role in BPS-induced hepatocellular injury (Maus et al., 2023). System Xc^−^ is an amino acid transporter protein complex composed of SLC7A11 and SLC3A2, responsible for maintaining intracellular redox homeostasis (Cao et al., 2025). BPS exposure may reduce intracellular glutathione (GSH) synthesis by inhibiting System Xc^−^ activity, thereby leading to lipid peroxidation and ferroptosis. However, it remains unclear whether exposure to BPS during pregnancy and lactation can induce hepatic metabolic dysfunction in offspring and ferroptosis, thereby exerting hepatotoxic effects.

Therefore, we established a mouse model of perinatal exposure to BPS to investigate whether the multigeneration liver toxicity of BPS is associated with hepatic lipid metabolic disorders and ferroptosis. Hematoxylin and eosin (H&E) and Oil Red O staining were used to analyze the liver injury and lipid accumulation of offspring mice. As well as the alanine aminotransferase (ALT) and aspartate aminotransferase (AST) were used to assess the function of liver. Mitochondrial ultrastructure, iron content and oxidative stress were used to observe the ferroptosis state. At the same time, we investigate the expression of proteins and mRNA level of which related to lipid synthesis, fatty acid oxidation as well as ferroptosis. Meanwhile, we established an *in vitro* model to verify the role and mechanism of ferroptosis related lipid metabolism abnormalities in BPS induced hepatotoxicity. This study aims to reveal the potential effects of BPS exposure during pregnancy and lactation on the liver metabolic function of offspring mice, especially the mechanism by which it induces ferroptosis by disrupting liver lipid metabolism. It provides a new perspective for understanding how environmental chemicals affect the health of their offspring through the mother.

## 2 Materials and methods

### 2.1 Chemicals and kits

4,4.-Sulfonyldiphenol (BPS, 98%) was purchased from Sigma-Aldrich (Shanghai) Trading Co.,Ltd. (#103039-100g). Mouse ALT, AST, TG, total cholesterol (T-CHO), high-density lipoprotein cholesterol (HDL-C), low-density lipoprotein cholesterol (LDL-C), (C009-2-1, C010-2-1, A110-1-1, A111-1-1, A112-1-1, A113-1-1) and Superoxide dismutase (SOD), catalase (CAT), malondialdehyde (MDA), reduced GSH kits (A001-3, A007-1-1, BC0025, A006-2-1), intracellular Iron Colorimetric Assay Kit (A039-2-1)were all obtained from Nanjing Jiancheng Institute of Bioengineering (Nanjing, China).

### 2.2 Animals and treatment

8 weeks female and male C57BL/6J mice (18–22 g) were purchased from (China Hunan Slake Jinda Animal Co. Ltd. (SCXK (Xiang) 2019–0004). Animals were housed individually in the animal care facility and maintained in a regular light cycle (12 h light/dark photoperiod) and at 20 °C–22 °C room temperature, with *ad libitum* access to food and water. Following a 1-week acclimatization period, two females in proestrus were paired with a fertile male overnight. The appearance of a copulatory plug is recorded as the gestational day 0 (GD0). Pregnant mice were housed individually and randomly assigned to four groups (n = 12 per group). The control group received and the experimental groups received. All the mice treated with drinking water with 0.1% anhydrous ethanol (Control group) or different concertration BPS (0.2, 2, or 20 mg/L BPS group), which was first dissolved in 1 mL of anhydrous ethanol and then added to 999 mL of drinking water from GD0 to postnatal day 21 (PD21). Offspring mice were maintained under the same housing conditions until PD28. At the end of the treatment period, mice were euthanized after intraperitoneal injection of pentobarbital sodium (40 mg/kg). Blood and organs were collected for subsequent experiments. All animal experiments comply with the ARRIVE guidelines. We also confirm that all animal experimental procedures conducted in this study were strictly performed in accordance with the ethical guidelines of the European Union Directive 2010/63/EU on the protection of animals used for scientific purposes, and were approved by the Institutional Animal Care and Use Committee at Guilin Medical University (Ethical approval number: GLMU-IACUC-2025–1077).

### 2.3 Liver steatosis evaluation and histological analysis

Freshly isolated mouse liver tissues (n = 3) fixed in 10% neutral buffered formalin at 4 °C and observe the hepatic lipid deposition after BPS exposure by Oil Red O staining according to the standard protocols (G1262, Solarbio, Beijing, China). Following the instructions of the modified Oil Red O kit, the liver was embedded and sectioned into 8-μm-thick frozen sections. The slides with adsorbed liver tissue sections were then placed in 60% isopropanol for 2 min, briefly rinsed in iced water (ice-water mixture), stained with Oil Red O solution for 10 min (protected from light), briefly immersed in 60% isopropanol for 5 s, and washed with iced water. Cell nucleus were subsequently stained with hematoxylin for 5 min. The edges of the tissues were dried after washed with ice water. Finally, glycerol gelatin was used to seal the sections, and photographs were obtained under an inverted microscope. Freshly collected liver samples (n = 3) were rinsed, dried, fixed with 4% paraformaldehyde and embedded in paraffin. Liver sections were deparaffinized with xylene (twice for 10 min each), anhydrous ethanol (twice for 5 min each) and then rehydrated through a graded ethanol series (95%, 90%, 80%, 70%) and water. The sections were then stained with hematoxylin for 5 min to visualize nucleus, rinsed with running water and counterstained with eosin for 1 min to visualize cytoplasm. Subsequently, the sections were dehydrated in anhydrous ethanol three times (5 min each), cleared with xylene three times (10 min each) and mounted with neutral adhesive. Representative sections were imaged using an inverted microscope.

### 2.4 Cell culture and viability analysis

The AML12 mouse hepatocyte cells were cultured in DMEM (Gibco, Waltham, MA, United States) supplemented with 10% fetal bovine serum (FBS) and 1% penicillin-streptomycin at 37 °C with 5% CO_2_. Cells were divided into four groups: control (CON), BPS (200 μM), BPS + Fer-1 (1 μM), and Fer-1 alone. Cells in 96-well plates were treated with BPS concentrations from 0 to 400 μM for 24 h, with or without Fer-1 pretreatment. After treatment, cells were incubated with CCK-8 reagent (1:9 in medium) for 2 h at 37 °C, and absorbance was measured at 450 nm. Based on the results, 200 μM BPS (75% viability) was selected for subsequent experiments.

### 2.5 Morphology of hepatocyte mitochondria by transmission electron microscopy

The liver tissues of three mice for each group were randomly selected and fixed with 2.5% glutaraldehyde for 6 h, followed by 1% osmium tetroxide for 1 h. The fixed tissues were dehydrated through a graded acetone series and embedded in epoxy resin. The embedded tissue blocks were cut into 70 nm slices by an ultramicrotome. Following staining with 4% uranyl acetate and lead citrate, mitochondrial morphology was examined using a transmission electron microscope (HT7800, Hitachi, Japan). Mitochondrial number, diameter, and cross-sectional area were measured using ImageJ software.

### 2.6 Evaluate liver function and oxidative damage

In this study, serum biochemical indices including ALT, AST, TG, T-CHO, HDL-C, LDL-C were all detected according to the manuscript. Meanwhile, the liver tissues were homogenized with 0.9% NaCl solution and centrifuged to obtain the supernatant (2500 r/min, 10 min). AML12 cells were washed with phosphate-buffered saline (PBS) after treated BPS and Fer-1 for 24 h. And then homogenization by dounce homogenizer on ice. The levels of SOD, CAT, MDA, GSH in tissues were used to reflect the oxidative damage level in the liver and AML12 cells. And all the kits were used a full-wavelength scanning multifunction microplate reader (Thermo Scientific Varioskan LUX, Waltham, MA, United States). Intracellular ROS levels in AML12 cells were measured using the fluorescent probe DCFH-DA. After treatment, cells in 6-well plates were washed with PBS and incubated with 10 μM DCFH-DA in serum-free medium at 37 °C for 20 min in the dark. Following two washes with warm serum-free medium, fluorescence intensity was immediately detected using a fluorescence microscope (excitation 488 nm, emission 525 nm).

### 2.7 Liver iron content detection

Fresh liver tissue was homogenized with 0.9% NaCl solution, and centrifuged at 2500 r/min, 10 min. Supernatant and AML12 cell homogenates were used to analyze the Fe^2+^ content. Absorbance measurements were conducted at 550 nm using a (Thermo Scientific Varioskan LUX, Waltham, MA, United States) spectrophotometer according to the manufacturer’s protocol. A standard curve was generated using serial dilutions of iron standards (0–100 μM), from which iron ion concentrations were determined through linear regression analysis (R^2^ > 0.99).

### 2.8 RNA extraction and RT-qPCR

Mouse liver tissues and AML12 cells were washed with cold phosphate-buffered saline (PBS), and total RNA was extracted using TRIzol reagent (Ambion, Carlsbad, CA, United States). RNA concentration and purity were determined using a NanoDrop 2000 spectrophotometer (Thermo Fisher Scientific, Waltham, MA, United States). Reverse transcription of 1 μg total RNA was performed using a first-strand cDNA synthesis kit (Takara Bio Inc., Beijing, China). Quantitative real-time PCR (RT-qPCR) was performed using SYBR® TB Green Premix Ex Taq (Takara Bio Inc.) to quantify the expression levels of target genes, including *TNF-α, IL-1β, CD36, Cpt1b, SREBP1C, PPARα, SCD1, FASN, GPX4, ACSL4, PTGS2, SLC7A11, HO-1*, and *TfR1*. Relative expression levels of target genes were calculated using the 2^−ΔΔCT^ method. The primer sequences are listed in Table 1.

**TABLE 1:** Primer sequences for real-time quantitative polymerase chain reaction.

Gene	Primer	Sequences (5′→ 3′)
*TNF-α*	Forward	ACGCTCTTCTGCCTGCTG
	Reverse	CTTGTCACTCGGGGTTCG
*IL-1β*	Forward	GAT​GGC​TTA​TTA​CAG​TGG​C
	Reverse	TAGTGGTGGTCGGAGATT
*CD36*	Forward	ATTCTCATGCCAGTCGGA
	Reverse	TTTGCTGCTGTTCTTTGC
*Cpt1b*	Forward	AGACTGTGCGTTCCTGTA
	Reverse	TTGGAGACGATGTAAAGG
*SREBP1C*	Forward	TCTCCTAGAGCGAGCGTT
	Reverse	AGGGCATCTGAGAACTCC
*PPARα*	Forward	CAAGTGCCTGTCTGTCGG
	Reverse	CAGGTAGGCTTCGTGGAT
*SCD1*	Forward	CTC​TTC​GGG​ATT​TTC​TAC​T
	Reverse	CCTTGTAAGTTCTGTGGC
*FASN*	Forward	CGGAGTCGCTTGAGTATA
	Reverse	CACAGGGACCGAGTAATG
*GPX4*	Forward	ATTCTCAGCCAAGGACAT
	Reverse	CAGGATTCGTAAACCACA
*ACSL4*	Forward	ACT​TTC​CAC​TTG​TGA​CTT​TAT
	Reverse	CTTCAGTTTGCTTTCCAG
*PTGS2*	Forward	ATCAGGTCATTGGTGGAG
	Reverse	ACACTCTGTTGTGCTCCC
*SLC7A11*	Forward	TTG​GAG​CCC​TGT​CCT​ATG​C
	Reverse	CGA​GCA​GTT​CCA​CCC​AGA​C
*HO-1*	Forward	CCCTGGAAGAGGAGATAG
	Reverse	GTG​GAG​ACG​CTT​TAC​ATA​G
*TfR1*	Forward	GAG​TGG​CTA​CCT​GGG​CTA​T
	Reverse	TGTCTGTCTCCTCCGTTT
*GAPDH*	Forward	CCT​CGT​CCC​GTA​GAC​AAA​ATG
	Reverse	TGA​GGT​CAA​TGA​AGG​GGT​CGT

### 2.9 Western blotting

Liver tissues and AML12 cells were mixed with tissue protein lysis buffer (Beyotime, Shanghai, China) containing 1 mM phenylmethane sulfonyl fluoride (PMSF, Beyotime Biotechnology, ST506). The total protein was extracted and the protein concentration was determined using a Bicinchoninic acid (BCA) kit (Beyotime, Shanghai, China). The proteins were denatured after centrifugation and the protein was loaded onto sodium dodecyl sulfate-polyacrylamide gels (10%) for electrophoretic separation and then transferred onto polyvinylidene fluoride (PVDF, IPVH00010, servicebio, Wuhan, China). After blocking with 5% skim milk in PBS-buffered saline containing 0.1% Tween 20 (PBST), the membranes were incubated overnight at 4 °C with primary antibodies targeting human leukocyte differentiation antigen 36 (CD36), carnitine palmitoyltransferase 1b (Cpt1b), sterol regulatory element-binding protein 1c (SREBP1C), peroxisome proliferator-activated receptor α (PPARα), glutathione peroxidase 4 (GPX4), long-chain acyl-coa synthetase 4 (ACSL4), and transferrin receptor 1 (TfR1). The membranes were washed three times with PBST, then they were incubated with secondary antibodies (Servicebio, Wuhan, China) for 1 h at room temperature. Finally, detected the expression of the target protein by enhanced chemiluminescence reagent and chemiluminescence imaging system obtained immunoreactive protein bands were imaged.

### 2.10 Statistical analysis

Data is expressed as mean ± standard error of mean (SEM). Statistical significance was defined as *P*<0.05. Statistical significance was identified by analysis of variance (ANOVA) followed by the least significant difference (LSD) test. When the data did not meet the normality and homogeneity of variance, Kruskal–Wallis analysis was used to compare the differences between the groups. All statistical analyses were performed using GraphPad Prism version 9.0 (GraphPad Software, San Diego, CA, United States).

## 3 Results

### 3.1 Ferroptosis is involved in BPS induced hepatotoxicity in mice

**FIGURE 1:**
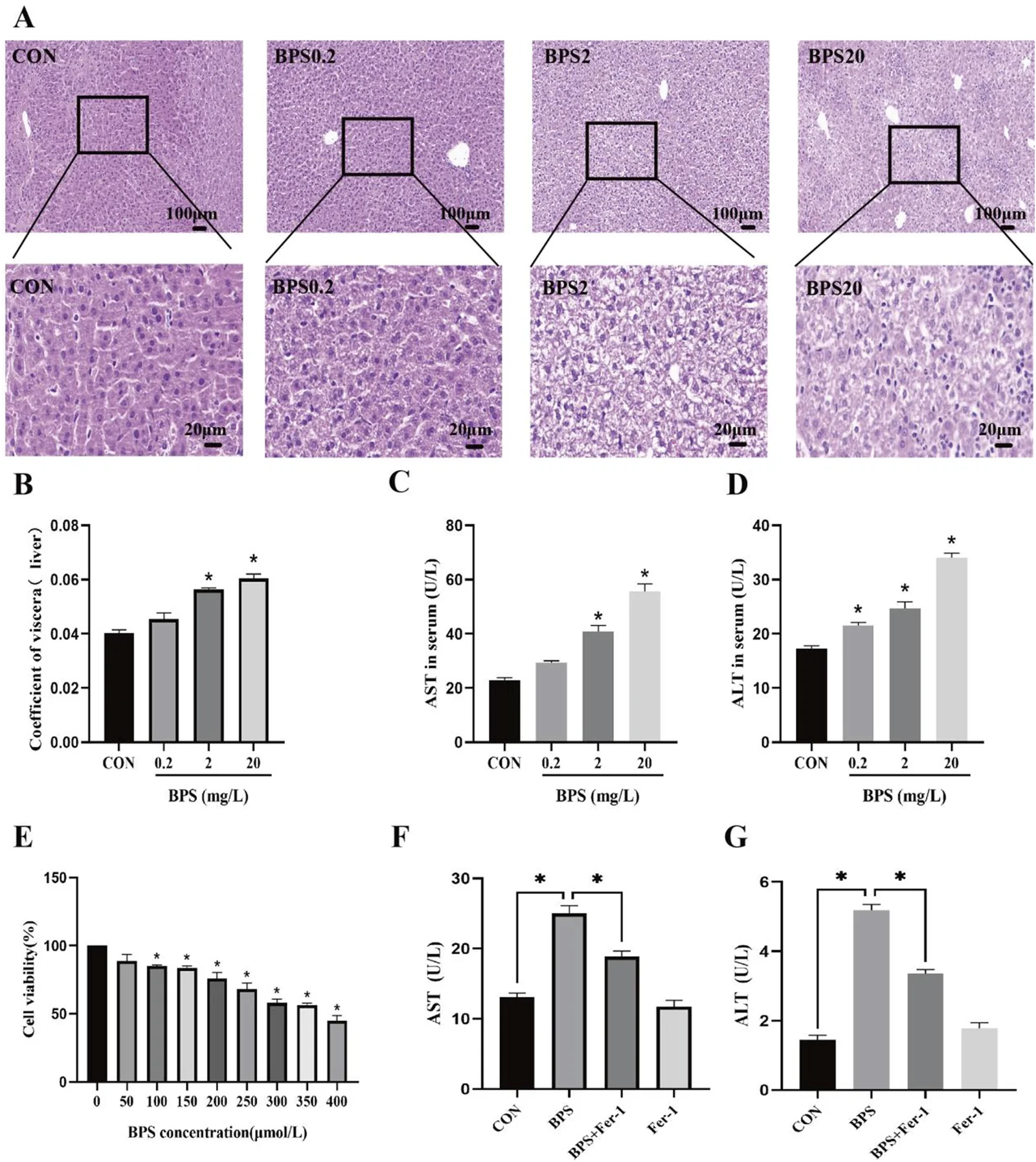
BPS caused liver injury and dysfunction. **(A)** The morphology of liver subjected to H&E staining. **(B)** Liver organ coefficient (n = 10, *P < 0.05). **(C,D)** AST and ALT levels of serum. (n = 6, *P < 0.05 vs. Control (CON). **(E)** Effects of different concentrations of BPS on the viability of AML12 cells (n = 3, *P < 0.05). **(F,G)** AST and ALT levels of AML12 cells. (n = 6,*P < 0.05).

To investigate whether gestational and lactational exposure to BPS had any toxic effect on the liver of offspring mice, liver organ coefficient analysis, H&E staining of the liver, and the level of ALT and AST in the serum were used to analyze the morphology and function of the liver. The liver organ coefficient of offspring mice was significantly increased (Figure 1B). H&E staining result revealed that hepatocytes in the control and 0.2 mg/L BPS groups were neatly arranged with uniform cytoplasm and centrally located nuclei, exhibiting no evident structural damage or significant hemorrhagic points. But in the 2 mg/L and 20 mg/L BPS group, liver cells exhibited disorganized arrangement, irregular sizes, and increased bleeding points, accompanied by inflammatory infiltration (Figure 1A). At the same time, a significant dose-dependent increase in ALT and AST levels was observed in the BPS treatment group (Figure C and D). At the same time, the CCK-8 assay revealed a significant, dose-dependent decrease in AML12 cells viability following BPS exposure (Figure E). Consistently, the activity levels of the liver function enzymes ALT and AST were significantly elevated in the BPS-treated groups compared to the control in the offspring mice and AML12 cells. However, the level of ALT and AST in the AML12 cells were significantly decreased after treated by Fer-1 (Figure F and G). All the above results indicate that BPS lead to hepatotoxic effects, and the hepatic dysfunction induced by BPS may be associated with ferroptosis.

### 3.2 BPS induces ferroptosis in the liver

**FIGURE 2:**
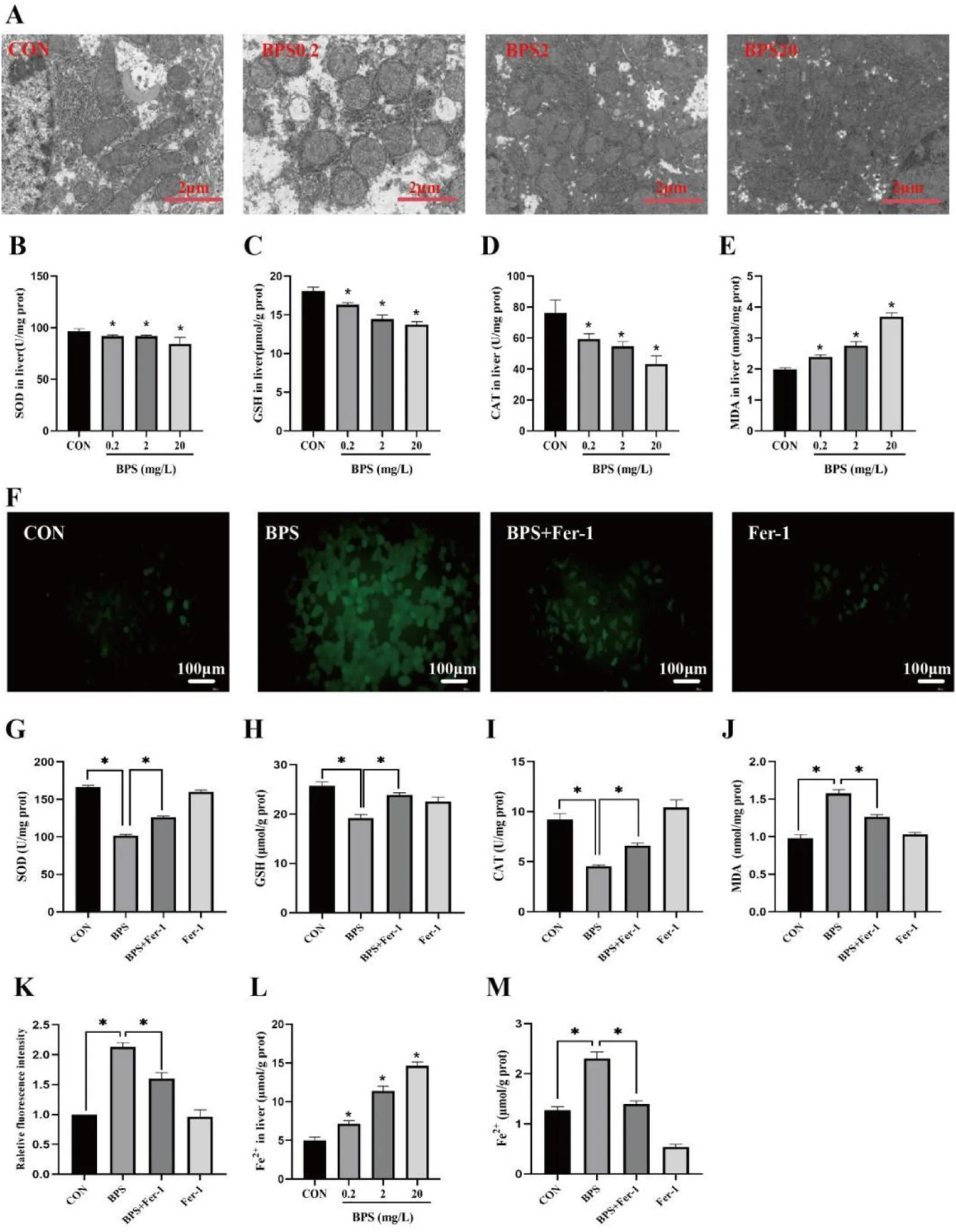
BPS induces ferroptosis in the liver. **(A)** The ultrastructure of liver mitochondria. Scale bars = 2 μm. **(B–E)** BPS changed the indicators of hepatic antioxidant of mouse live tissues (n = 6, *P < 0.05). **(F,K)** The fluorescence intensity of ROS in the AML12 cells (×200 magnification). **(G–J)** BPS changed the indicators of hepatic antioxidant of AML12 cells (n = 6, *P < 0.05). **(L)** The Fe^2+^ levels in the liver tissues (n = 6, *P < 0.05 vs. CON). **(M)** The Fe^2+^ levels in the AML12 cells (n = 6, *P < 0.05).

To determine whether BPS induced the ferroptosis in the offspring mice liver. We tested oxidative stress related indicators and found that the activities of SOD, GSH, and CAT were decreased, while the level of MDA was significantly increased in the offspring mice which treated by BPS (Figures 2B–E). As well as, transmission electron microscopy (TEM) was employed to examine the ultrastructure of the hepatocytes, and the result showed that hepatocytes exhibited pronounced structural alterations such as characteristic morphological changes, including condensed membranes with increased electron density, marked swelling and rounding, fragmentation or complete loss of cristae, and disruption of the outer membrane (Figure 2A). These morphological alterations exhibited a clear BPS dose-dependent response. At the same time, we quantified iron concentration in liver tissue, and the result indicated that BPS treatment significantly increased hepatic iron levels in all experimental groups compared to the control (Figure 2L). Consistent with the *in vivo* findings, the activities of key antioxidant enzymes, including SOD, GSH, and CAT were decreased but increased by Fer-1, while MDA levels were markedly elevated in the BPS-treated group but decreased in BPS and Fer-1 group (Figures 2G–J). The effect of BPS on intracellular ROS levels was assessed in AML12 cells *in vitro*. A pronounced increase in fluorescence intensity was observed in the BPS-treated group, which was significantly attenuated by Fer-1 co-treatment (Figure 2F). Furthermore, compared to the control, intracellular iron content was significantly higher in BPS group, but Fer-1 also decreased the level of iron concertration in AML12 cells (Figure 2M). These results indicate that BPS exposure causes oxidative damage in the liver and hepatocytes, increases iron content.

### 3.3 BPS altered the expression of ferroptosis-related genes

**FIGURE 3:**
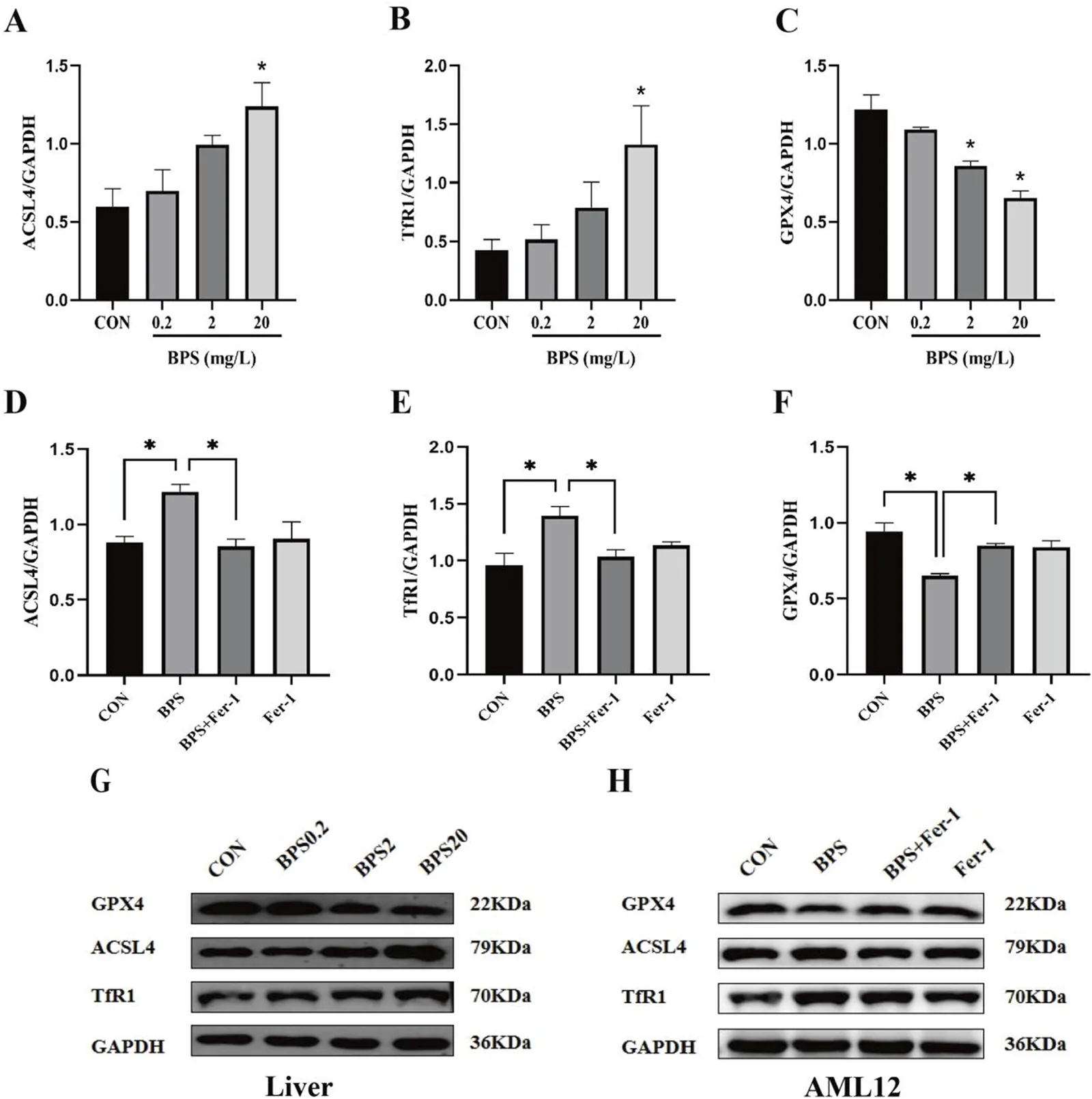
BPS altered the expression of ferroptosis-related genes. **(A–C)** Western blot detection of ferroptosis-related protein expression in liver tissue exposed to BPS (n = 3, *P < 0.05 vs. CON). **(D–F)** Western blot detection of ferroptosis-related protein expression in AML12 cells exposed to BPS and Fer-1 (n = 3, *P < 0.05). **(G)** The protein levels of GPX4, ACSL4, and TfR1 in liver tissues (n = 3, *P < 0.05 vs. CON). **(H)** The protein levels of GPX4, ACSL4, and TfR1 in AML12 cells (n = 3, *P < 0.05).

At the same time, we analyzed the mRNA expression levels of ferroptosis-related genes. These results showed that the expression levels of *GPX4, Nrf2, SLC7A11*, and *HO-1* were dramatically suppressed (Supplementary Figure S1A-D). Conversely, the levels of genes associated with iron metabolism (*ACSL4, PTGS2*, and *TfR1*) were elevated (Supplementary Figure S1E-G). We further verified the protein expression levels of ferroptosis-related indicators including GPX4, ACSL4, and TfR1 by western blot analysis and these changes were consistent with the RT-qPCR results. Compared to the control group, the protein expression levels of ACSL4 and TfR1 were increased (Figures 3A,B), but GPX4 was decreased in each BPS dose group (Figure 3C). These changes were also indicated *in vitro* cell experiments. The mRNA expression of key antioxidant and ferroptosis-suppressive genes, including *GPX4, Nrf2, SLC7A11*, and *HO-1*, was significantly downregulated in the BPS-treated group compared to the control (Supplementary Figure S2A-D). Conversely, the expression of pro-ferroptosis markers associated with iron metabolism and lipid peroxidation-such as *ACSL4*, *PTGS2*, and *TfR1* were was markedly upregulated (Supplementary Figures S2E-G). These transcriptional alterations were further confirmed at the protein level by western blot analysis, demonstrating a consistent pattern of expression across both mRNA and protein levels (Figures 3D–H). At the same time, Fer-1 significantly reversed the BPS-induced changes in ferroptosis-related proteins, including GPX4, ACSL4, and TfR1. Collectively, these findings demonstrate that BPS exposure significantly induces ferroptosis in the hepatocytes.

### 3.4 BPS induced hepatic ferroptosis is associated with abnormal lipid metabolism

**FIGURE 4:**
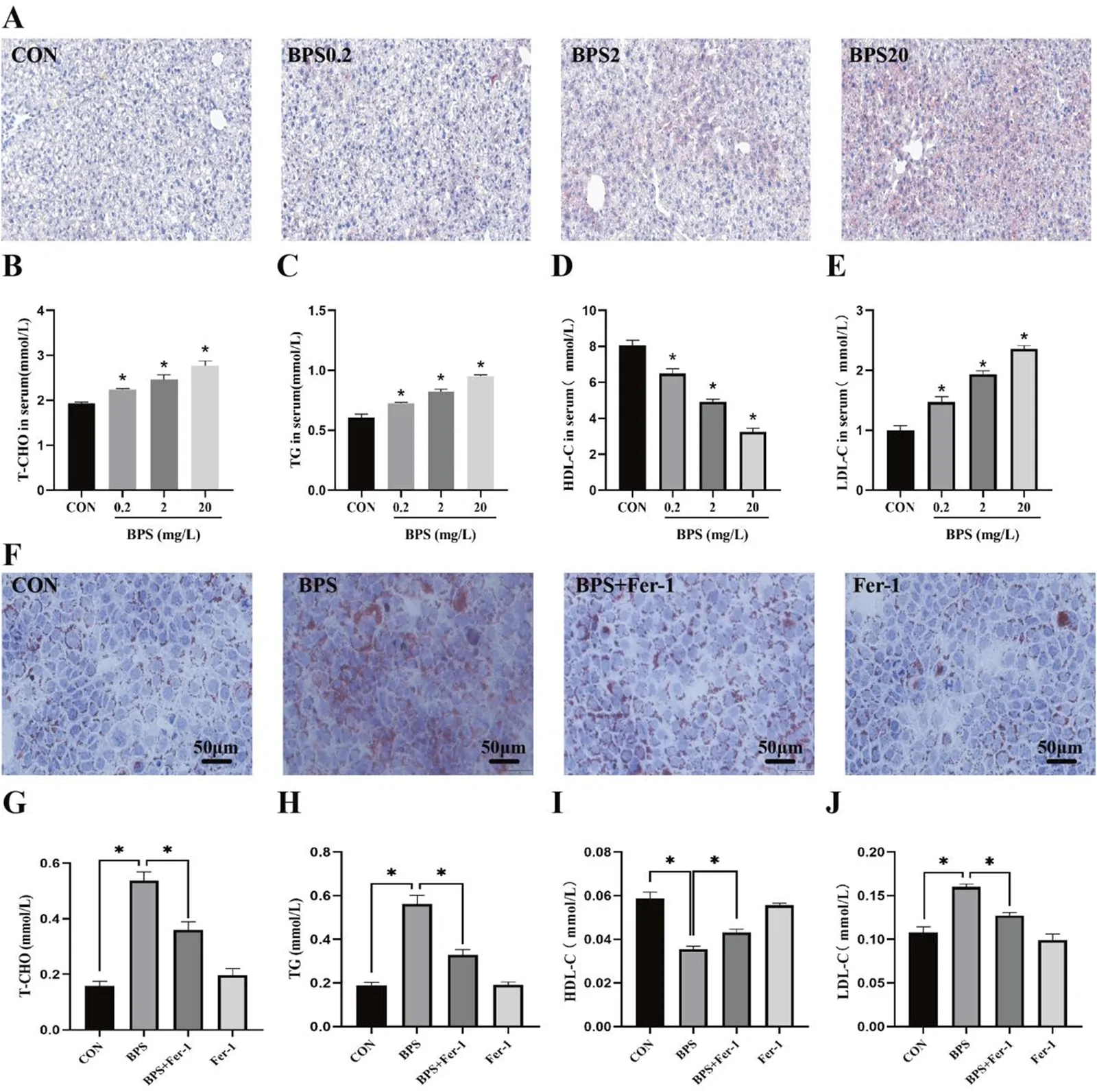
BPS-induced hepatic ferroptosis is associated with abnormal lipid metabolism. **(A)** Oil Red O staining of liver tissue (×200magnification). **(B–E)** T-CHO, TG, HDL-C, and LDL-C levels in serum. (n = 6, *P < 0.05 vs. CON). **(F)** Oil Red O staining of AML12 cells (×400magnification). **(G–J)** The levels of T-CHO, TG, HDL-C, and LDL-C in AML12 cells (n = 6, *P < 0.05).

In order to clarify the role of lipid metabolism disorder in BPS-induced liver injury, Oil Red O staining was used to analyze the hepatic lipid accumulation. Compared to the control group, BPS increased numerous lipid droplets in liver tissues (Figure 4A). In addition, the levels of several lipid metabolism-relevant parameters were assessed. As Figures 4B–E, BPS exposure significantly increased serum levels of total cholesterol (TC), triglycerides (TG), and low-density lipoprotein cholesterol (LDL-C), while decreasing high-density lipoprotein cholesterol (HDL-C). Consistent with these findings, *in vitro* experiments demonstrated that cells in the BPS-treated group exhibited a significant increase in lipid droplet accumulation (Figure 4F), alongside dyslipidemia characterized by TC, TG, and LDL-C, as well as reduced HDL-C, however, lipid accumulation and abnormal lipid metabolism induced by BPS were both restored after Fer-1 treatment (Figures 4G,H). These results indicate that BPS-induced alterations in hepatic lipid synthesis, transport, and fatty acid oxidation are influenced by ferroptosis.

### 3.5 Fer-1 ameliorates BPS induced alterations in lipid metabolism-related genes

**FIGURE 5:**
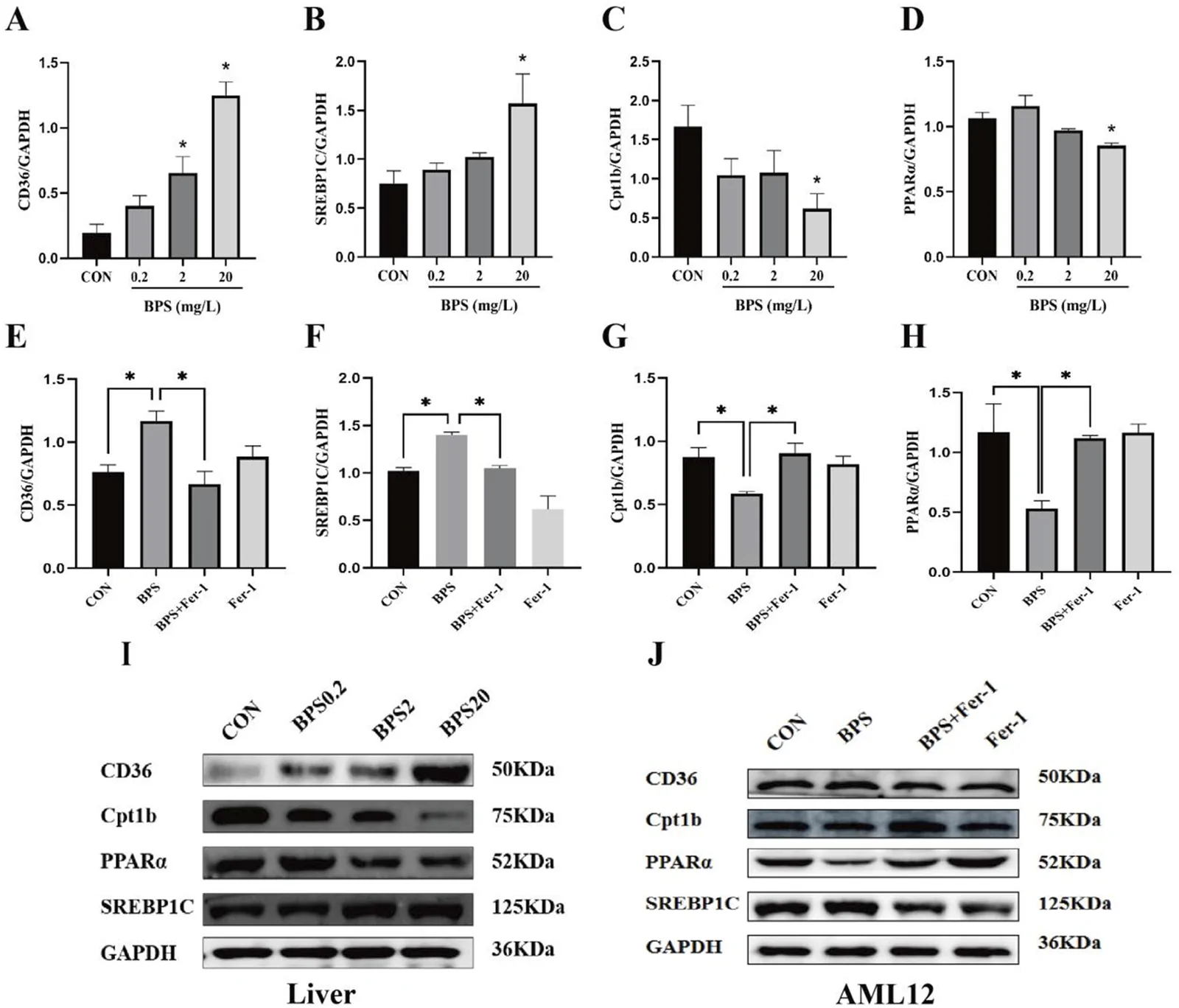
Western blot used to analysis the protein expression level of hepatic lipid metabolism-related protein in liver tissue and AML12 cells after BPS exposure. **(A–D)** The protein expression level in livers exposed to BPS (n = 3, *P < 0.05 vs. CON). **(E–H)** The protein expression level in AML12 cells exposed to BPS (n = 3, *P < 0.05). **(I)** The protein levels of CD36, Cpt1b, SREBP1C, and PPARα in liver tissues (n = 3, *P < 0.05 vs. CON). **(J)** The protein levels of CD36, Cpt1b, SREBP1C, and PPARα in AML12 cells treated by BPS (n = 3, *P < 0.05).

Meanwhile, we also quantified the expression levels of key mRNA involved in lipid biosynthesis, transport and fatty acid oxidation. These results showed that compared to the control group, mRNA expression levels of TNF-α, IL-1β, CD36, SREBP1C, SCD1, and FASN were significantly upregulated (Supplementary Figures S3A-F). In contrast, mRNA expression levels of PPARα and Cpt1b were downregulated (Supplementary Figures S3G,H). *In vitro* experiments further demonstrated that the mRNA expression of pro-inflammatory cytokines (TNF-α, IL-1β), the fatty acid transporter CD36, and key lipogenic genes (SREBP1c, SCD1, FASN) was significantly upregulated in BPS-treated cells (Supplementary Figures S3A-F). Conversely, the expression of genes involved in fatty acid oxidation, including PPARα and CPT1b, was markedly downregulated (Supplementary Figures S3G, H). However, the mRNA level of all the above-mentioned genes were recovered by Fer-1 (Supplementary Figures S4A-D). At the same time, western blot results also indicated that BPS increased the protein expression levels of CD36 and SREBP1C (Figures 5A,B), while decreased the expression protein levels of Cpt1b and PPARα (Figures 5C,D) and these changes were consistent with the RT-qPCR results. However, in the AML12 cell model, co-exposure to BPS and Fer-1 significantly reduced CD36 and SREBP-1c protein levels and increased PPARα and CPT1b expression compared with the BPS group (Figures 5E–H). These results indicated that BPS caused liver lipid metabolism disorders.

## 4 Discussion

Although BPS has been widely adopted as a substitute for BPA, many studies have not adequately assessed the potential for BPS exposure to induce adverse metabolic effects (Lin et al., 2025). Epidemiological investigations have consistently linked BPS exposure to hepatotoxicity in human populations (Li C.-L. et al., 2025). In this study, pregnancy and lactation exposure to BPS significantly promote the lipid accumulation in liver tissue of offspring mice by triggering ferroptosis. These phenomena can be reversed in cell experiments using ferroptosis inhibitors. In the present study, our findings provide evidence that BPS induced lipid metabolic dysregulation and ferroptosis in hepatocytes.

There are increasing evidences showed that BPA induces hepatic pathological alterations and disrupts lipid metabolism (Azevedo et al., 2019; He et al., 2023). In our study, BPS exposure increased the organ coefficient of liver, but also the histological staining further confirmed that BPS induced the hepatic pathological alterations like increase the number of inflammatory cells in the liver, which was consistent with previous reports of perinatal bisphenol exposure in mice by Meng et al. (2019). At the same time, BPS lead to the increasing of ALT and AST as well as the expression of IL-1β and TNF-α in offspring mice, which proved the occurrence of liver inflammation and injury were upregulated. Moreover, BPS has been shown to promote the release of IL-1β in the liver (Qin et al., 2020). Interestingly, abnormalities in cholesterol and LDL-C metabolism also lead to dysregulation of inflammasome components, resulting in the generation of IL-1β and IL-18 (Wani et al., 2021). Previous studies have demonstrated that BPS disrupts hepatic lipid metabolism through oxidative stress and mitochondrial dysfunction (Azevedo et al., 2019). The level of TG, T-CHO, and LDL-C in the BPS dose groups were increased, and the content of HDL-C was decreased, indicating dysregulation of lipid metabolism in offspring mice. These findings were further supported by Oil Red O staining, which revealed increased lipid droplet accumulation in the liver. SREBP1C is an insulin-stimulated FA synthesis-stimulating transcription factor that increases the transcription of genes encoding FASN and SCD1 and that regulates plasma TG levels (Li N. et al., 2023). Our study showed elevated expression levels of genes involved in hepatic lipid synthesis (SREBP1C, SCD1 and FASN) compared to controls. Increases in SREBP1C and its target genes result in elevated TG levels in adipocytes and lead to lipid accumulation. Expression levels of a gene involved in fatty acid uptake (CD36), which is associated with FA transport and uptake (Wasityastuti et al., 2025) and is responsive to ROS levels (Kim et al., 2017), are also elevated. Reduced expression levels of genes involved in hepatic fatty acid oxidation (PPARα and Cpt1b), in turn, reduced lipid oxidation. This transcriptional pattern was mirrored at the protein level. Collectively, these findings suggest that BPS exposure induces hepatic lipid accumulation through concurrent upregulation of lipid synthesis, enhanced fatty acid uptake, and suppressed fatty acid oxidation, ultimately leading to lipid metabolism disorders accompanied by inflammatory responses.

However, our results suggest that BPS-induced abnormal lipid synthesis appears to be influenced by ferroptosis inhibition (Fer-1). Ferroptosis is primarily driven by oxidative damage resulting from mitochondrial dysfunction and the excessive accumulation of iron-dependent lipid peroxidation products (Zhang X. et al., 2024). In our study, BPS exposure induced hepatic oxidative stress, as evidenced by increased MDA levels and significantly reduced activities of CAT, GSH, and SOD. These findings are consistent with previous reports (Lv et al., 2022; Wu et al., 2023). Meanwhile, significant iron accumulation and severe mitochondrial damage, including reduced or absent cristae, mitochondrial membrane condensation, and vacuolation, were observed in the livers of BPS-exposed mice. TfR1 is frequently upregulated in rapidly dividing cells and is essential for meeting the iron requirements of cells during growth. Accumulation of TfR1 at the cell surface in the presence of ferroptosis is thought to be a distinguishing feature (Jing et al., 2024). Our result indicated that BPS significantly increased the expression of TfR1. GPX4 is a key regulator of ferroptosis which had been demonstrated that decreased GPX4 expression enhances ferroptosis, while GPX4 over expression suppresses ferroptosis (Lv et al., 2022; Zhang W. et al., 2024). SLC7A11 is essential for maintaining redox homeostasis by facilitating GSH synthesis. The SLC7A11 downregulation may lead to disruption of the Xc-system which in turn can lead to cysteine deficiency, GSH depletion and ultimately ferroptosis (Wang et al., 2020). ACSL4 was also recognized as a key regulator of ferroptosis, there is research showed that knockout of ACSL4 in ferroptosis-sensitive mouse and human cells abolishes protection against cell death (Lee and Gan, 2022). At the same time, PTGS2 plays a critical role in inflammatory responses, cell proliferation, apoptosis, and other pathological processes, highlighting its importance in ferroptosis biology (Xie et al., 2016). In our result, the expression levels of GPX4, HO-1 and SLC7A11 were significantly reduced, whereas the expression levels of ACSL4, PTGS2 were significantly increased in the offspring mice which treated by BPS. In all, BPS enhances iron deposition and contributes to lipid peroxidation, thereby promoting ferroptosis.

To further verify whether BPS-induced abnormal lipid metabolism is affected by ferroptosis, we established an *in vitro* co-exposure model of BPS and Fer-1. In our study, BPS significantly increased the level of ROS and MDA, but decreased the content of CAT and SOD as well as GSH. Iron content detection revealed a significant increase in intracellular iron accumulation, which was consistent with the characteristic of hepatic iron accumulation *in vivo*. At the same time, BPS treatment significantly downregulated ferroptosis-inhibiting molecules (GPX4, HO-1, SLC7A11) and upregulated ferroptosis-promoting molecules (ACSL4, PTGS2, TfR1), consistent with *in vivo* observations. Majority of research studies have shown that Fer-1 can attenuate oxidative damage and alleviate the onset of ferroptosis (Liang et al., 2024; Sun et al., 2024; Zhu et al., 2025). Our results also investigated that, compared with BPS group, the Fer-1 significantly restored oxidative damage, reduced lipid droplet accumulation, decreased iron content, and also upregulated the protein expression level of GPX4 but downregulated the protein expression level of ACSL4 and TfR1 in AML12 cells. These results demonstrate that BPS can induce ferroptosis in hepatocytes. There is a close and complex bidirectional regulatory relationship between ferroptosis and lipid metabolism. Lipid metabolism determines cellular sensitivity to ferroptosis by controlling the PUFAs/MUFAs ratio, lipid droplet metabolism, and energy status, while ferroptosis, in turn, influences lipid storage by promoting lipid oxidation and fatty acid metabolism (Shan et al., 2025; Sun et al., 2025). In our study, Oil Red O staining showed Fer-1 also alleviate the lipid droplet accumulation as well as changed the level of TG, T-CHO, LDL-C and HDL-C in the AML12 cells. At the molecular level, Fer-1 treatment elevated the protein levels of PPARα and Cpt1b but suppressed those of CD36 and SREBP1C compared to the BPS group. The concordance between *in vivo* animal studies and *in vitro* cellular experiments indicates that BPS exerts its hepatotoxic effects by multi-level regulation of ferroptosis and lipid synthesis.

## 5 Conclusion

Collectively, Pregnancy and lactation period exposure to BPS lead to obvious liver toxicity effects in offspring, our results elucidate the potential role and mechanism by which BPS promote the lipid accumulation and ferroptosis, thus leading to abnormal liver function. Notably, our study had certain limitations. Our research failed to directly explain the interaction between liver metabolic disorders and ferroptosis in offspring caused by BPS and liver toxic effects in the offspring. However, a co-exposure cellular model of a ferroptosis inhibitor and BPS was established in this study. This model elucidated the regulatory interplay between ferroptosis and lipid synthesis and their combined role in BPS induced hepatotoxicity. In all, this study is the first to establish ferroptosis as a novel mechanism in BPS-induced hepatotoxicity. These results provide critical evidence for the direct hepatotoxicity of BPS, offer new insights into its developmental risks, and establish a foundation for targeting ferroptosis or lipid metabolism in future interventions.

## Data Availability

The datasets presented in this study can be found in online repositories. The names of the repository/repositories and accession number(s) can be found in the article/Supplementary Material.

## References

[B1] Ahn Y.-A. Baek H. Choi M. Park J. Son S. J. Seo H. J. (2020). Adipogenic effects of prenatal exposure to bisphenol S (BPS) in adult F1 Male mice. Sci. Total Environ. 728, 138759. 10.1016/j.scitotenv.2020.138759 32403013

[B2] Azevedo L. F. Porto Dechandt C. R. Cristina de Souza R. C. Hornos C. M. F. Alberici L. C. Barbosa F. (2019). Long-term exposure to bisphenol A or S promotes glucose intolerance and changes hepatic mitochondrial metabolism in male Wistar rats. Food Chem. Toxicol. 132, 110694. 10.1016/j.fct.2019.110694 31344369

[B3] Blaauwendraad S. M. Voerman E. Trasande L. Kannan K. Santos S. Ruijter G. J. G. (2021). Associations of maternal bisphenol urine concentrations during pregnancy with neonatal metabolomic profiles. Metabolomics 17 (9), 84. 10.1007/s11306-021-01836-w 34518915 PMC8437833

[B4] Cao J. Zhou T. Wu T. Lin R. Huang J. Shi D. (2025). Targeting estrogen-regulated system x(c)(-) promotes ferroptosis and endocrine sensitivity of ER+ breast cancer. Cell Death Dis. 16 (1), 30. 10.1038/s41419-025-07354-0 PMC11756422 39833180

[B5] Dias G. R. M. Giusti F. C. V. de Novais C. O. de Oliveira M. A. L. Paiva A. G. Kalil-Cutti B. (2025). Intergenerational and transgenerational effects of endocrine-disrupting chemicals in the offspring brain development and behavior. Front. Endocrinol. 16, 1571689. 10.3389/fendo.2025.1571689 PMC12133532 40469448

[B6] Gebru Y. A. Pang M.-G. (2023). Modulatory effects of bisphenol A on the hepatic immune response. Environ. Pollut. 336, 122430. 10.1016/j.envpol.2023.122430 37611793

[B7] Hao K. Luo J. Sun J. Ge H. Wang Z. (2021). Associations of urinary bisphenol A and its alternatives bisphenol S and F concentrations with depressive symptoms among adults. Chemosphere 279, 130573. 10.1016/j.chemosphere.2021.130573 33878692

[B8] He W. Gao Z. Liu S. Tan L. Wu Y. Liu J. (2023). G protein-coupled estrogen receptor activation by bisphenol-A disrupts lipid metabolism and induces ferroptosis in the liver. Environ. Pollut. 334, 122211. 10.1016/j.envpol.2023.122211 37454720

[B9] Heinsberg L. W. Bui C. N. N. Hartle J. C. Sereika S. M. Choy C. C. Wang D. (2020). Estimated dietary bisphenol-A exposure and adiposity in samoan mothers and children. Toxics 8 (3), 67. 10.3390/toxics8030067 32887300 PMC7560430

[B10] Jing X. Wang W. He X. Liu X. Yang X. Su C. (2024). HIF-2α/TFR1 mediated iron homeostasis disruption aggravates cartilage endplate degeneration through ferroptotic damage and mtDNA release: a new mechanism of intervertebral disc degeneration. J. Orthop. Transl. 46, 65–78. 10.1016/j.jot.2024.03.005 PMC11130997 38808263

[B11] Kim D. H. Cho Y. M. Lee K. H. Jeong S. W. Kwon O. J. (2017). Oleate protects macrophages from palmitate-induced apoptosis through the downregulation of CD36 expression. Biochem. Biophys. Res. Commun. 488 (3), 477–482. 10.1016/j.bbrc.2017.05.066 28522296

[B12] Lee H. Gan B. (2022). Ferroptosis execution: is it all about ACSL4? Cell Chem. Biol. 29 (9), 1363–1365. 10.1016/j.chembiol.2022.08.002 36113403

[B13] Lee Y. J. Lim Y. H. Shin C. H. Kim B. N. Kim J. I. Hong Y. C. (2022). Relationship between bisphenol A, bisphenol S, and bisphenol F and serum uric acid concentrations among school-aged children. PLoS One 17 (6), e0268503. 10.1371/journal.pone.0268503 35709251 PMC9202957

[B14] Li N. Li X. Ding Y. Liu X. Diggle K. Kisseleva T. (2023a). SREBP regulation of lipid metabolism in liver disease, and therapeutic strategies. Biomedicines 11 (12), 3280. 10.3390/biomedicines11123280 38137501 PMC10740981

[B15] Li Z. Ru S. Li J. Yang Y. Wang W. (2023b). Continuous exposure to bisphenol S increases the accumulation of endogenous metabolic toxicants by obstructing the glucuronic acid pathway. Environ. Pollut. 336, 122433. 10.1016/j.envpol.2023.122433 37659633

[B16] Li C.-L. Yao Z.-Y. Zhang Y.-F. Cui X.-T. Sun A. Cao J.-Y. (2025a). Bisphenols exposure and non-alcoholic fatty liver disease: from environmental trigger to molecular pathogenesis. Front. Endocrinol. 16, 1606654. 10.3389/fendo.2025.1606654 PMC12137076 40475995

[B17] Li S. Fan Y. Tang M. Wu X. Bai S. Yang X. (2025b). Bisphenol S exposure and MASLD: a mechanistic Study in mice. Environ. Health Perspect. 133 (5), 57009. 10.1289/EHP17057 PMC12077661 40203079

[B18] Liang N. Song W. Li J. (2024). BPA promotes lung fibrosis in mice by regulating autophagy-dependent ferroptosis in alveolar epithelial cells. Ecotoxicol. Environ. Saf. 278, 116412. 10.1016/j.ecoenv.2024.116412 38691879

[B19] Lin K. X. Wu Z. Y. Qin M. L. Zeng H. C. (2025). Bisphenol S induces lipid metabolism disorders in HepG2 and SK-Hep-1 cells *via* oxidative stress. Toxics 13 (1), 44. 10.3390/toxics13010044 39853042 PMC11769282

[B20] Lv J. Hou B. Song J. Xu Y. Xie S. (2022). The relationship between ferroptosis and diseases. J. Multidiscip. Healthc. 15, 2261–2275. 10.2147/JMDH.S382643 36225859 PMC9549801

[B21] Maus M. López-Polo V. Mateo L. Lafarga M. Aguilera M. De Lama E. (2023). Iron accumulation drives fibrosis, senescence and the senescence-associated secretory phenotype. Nat. Metab. 5 (12), 2111–2130. 10.1038/s42255-023-00928-2 38097808 PMC10730403

[B22] Meng Z. Tian S. Yan J. Jia M. Yan S. Li R. (2019). Effects of perinatal exposure to BPA, BPF and BPAF on liver function in male mouse offspring involving in oxidative damage and metabolic disorder. Environ. Pollut. 247, 935–943. 10.1016/j.envpol.2019.01.116 30823348

[B23] Molangiri A. Varma S. M S. Kambham S. Duttaroy A. K. Basak S. (2022). Prenatal exposure to bisphenol S and bisphenol A differentially affects male reproductive system in the adult offspring. Food Chem. Toxicol. 167, 113292. 10.1016/j.fct.2022.113292 35842007

[B24] Peña de la Sancha P. Wieser B. I. Schauer S. Reicher H. Sattler W. Breinbauer R. (2025). Lipolysis-derived fatty acids are needed for homeostatic control of sterol element-binding protein-1c driven hepatic lipogenesis. Commun. Biol. 8 (1), 588. 10.1038/s42003-025-08002-1 40205023 PMC11982389

[B25] Peng H. Pfeiffer S. Varynskyi B. Qiu M. Srinark C. Jin X. (2025). Prion-induced ferroptosis is facilitated by RAC3. Nat. Commun. 16 (1), 5385. 10.1038/s41467-025-60793-3 40562790 PMC12198409

[B26] Qin J. Ru S. Wang W. Hao L. Ru Y. Wang J. (2020). Long-term bisphenol S exposure aggravates non-alcoholic fatty liver by regulating lipid metabolism and inducing endoplasmic reticulum stress response with activation of unfolded protein response in male zebrafish. Environ. Pollut. 263, 114535. 10.1016/j.envpol.2020.114535 32283406

[B27] Rezg R. Oral R. Tez S. Mornagui B. Pagano G. Trifuoggi M. (2022). Cytogenetic and developmental toxicity of bisphenol A and bisphenol S in Arbacia lixula sea urchin embryos. Ecotoxicology 31 (7), 1087–1095. 10.1007/s10646-022-02568-w 35838932 PMC9458557

[B28] Shan Y. Zhu X. Wang T. Zhang L. Qi Y. Hu Z. (2025). Mitochondria‐Targeted ferroptosis nanodrug for triple‐negative breast cancer therapy *via* Fatty acid metabolism remodeling and tumor bacterial symbiosis inhibition. Small 21 (39), e06443. 10.1002/smll.202506443 40823765

[B29] Sun Y. Sha M. Qin Y. Xiao J. Li W. Li S. (2024). Bisphenol A induces placental ferroptosis and fetal growth restriction *via* the YAP/TAZ-ferritinophagy axis. Free Radic. Biol. Med. 213, 524–540. 10.1016/j.freeradbiomed.2024.01.033 38326183

[B30] Sun D. Wang L. Wu Y. Yu Y. Yao Y. Yang H. (2025). Lipid metabolism in ferroptosis: mechanistic insights and therapeutic potential. Front. Immunol. 16, 1545339. 10.3389/fimmu.2025.1545339 40134420 PMC11932849

[B31] Tuzimski T. Pieniążek D. Buszewicz G. Teresiński G. (2018). QuEChERS-based extraction procedures for the analysis of bisphenols S and A in breast milk samples by LC-QqQ-MS. J. AOAC Int. 17. 10.5740/jaoacint.18-0297 30333076

[B32] Vandenberg L. N. Maffini M. V. Sonnenschein C. Rubin B. S. Soto A. M. (2009). Bisphenol-A and the great divide: a review of controversies in the field of endocrine disruption. Endocr. Rev. 30 (1), 75–95. 10.1210/er.2008-0021 19074586 PMC2647705

[B33] Wang L. Liu Y. Du T. Yang H. Lei L. Guo M. (2020). ATF3 promotes erastin-induced ferroptosis by suppressing system Xc. Cell Death Differ. 27 (2), 662–675. 10.1038/s41418-019-0380-z 31273299 PMC7206049

[B34] Wang J. Wu C. Zhang X. Song Y. Wang B. Zhang K. (2023). Developmental neurotoxic effects of bisphenol A and its derivatives in Drosophila melanogaster. Ecotoxicol. Environ. Saf. 260, 115098. 10.1016/j.ecoenv.2023.115098 37269611

[B35] Wani K. AlHarthi H. Alghamdi A. Sabico S. Al-Daghri N. M. (2021). Role of NLRP3 inflammasome activation in obesity-mediated metabolic disorders. Int. J. Environ. Res. Public Health 18 (2), 511. 10.3390/ijerph18020511 PMC7826517 33435142

[B36] Wasityastuti W. Tsany S. F. Pasaribu H. S. Saffana R. D. Wahyudi D. S. Nugrahaningsih D. A. A. (2025). Sulfo-N-Succinimidyl oleate sodium as CD36 inhibitor: dose optimization and its effects on FFA uptake, inflammation, and ER stress in HepG2 cells. J. Biochem. Mol. Toxicol. 39 (4), e70243. 10.1002/jbt.70243 40143623

[B37] Wu Z. Y. Luo L. Kan Y. Q. Qin M. L. Li H. T. He Q. Z. (2023). Puerarin prevents bisphenol S induced lipid accumulation by reducing liver lipid synthesis and promoting lipid metabolism in C57BL/6J mice. Toxics 11 (9), 736. 10.3390/toxics11090736 37755746 PMC10538013

[B38] Xie Y. Hou W. Song X. Yu Y. Huang J. Sun X. (2016). Ferroptosis: process and function. Cell Death Differ. 23 (3), 369–379. 10.1038/cdd.2015.158 26794443 PMC5072448

[B39] Xie C. Jiang X. Yin J. Jiang R. Zhu J. Zou S. (2025). Bisphenol S accelerates the progression of high fat diet-induced NAFLD by triggering ferroptosis *via* regulating HMGCS2. J. Hazard Mater. 487, 137166. 10.1016/j.jhazmat.2025.137166 39799675

[B40] Zhang W. Liu Y. Liao Y. Zhu C. Zou Z. (2024a). GPX4, ferroptosis, and diseases. Biomed. and Pharmacother. 174, 116512. 10.1016/j.biopha.2024.116512 38574617

[B41] Zhang X. Hu Y. Wang B. Yang S. (2024b). Ferroptosis: Iron-mediated cell death linked to disease pathogenesis. J. Biomed. Res. 38 (5), 1–23. 10.7555/JBR.37.20230224 PMC11461536 38808552

[B42] Zhu B. Sun C. Luo D. Liang Y. Jiang A. Jiang Z. (2025). Coptisine improves liver inflammation in sepsis by regulating STAT1/IRF1/GPX4 signaling‐mediated kupffer cells ferroptosis. Phytotherapy Res. 39 (9), 4308–4326. 10.1002/ptr.70063 40791017

