# Correction: Pregnancy and lactation exposure to bisphenol S induces ferroptosis via disrupted hepatic lipid metabolism in offspring mice

**DOI:** 10.3389/ftox.2026.1911506

**Published:** 2026-09-08

**Authors:** Mengfen Pan, Xinxin Guo, Caiyun Wei, Nuo Xu, Yan Su, Zhensong Ma, Huaicai Zeng

**Affiliations:** 1 Guangxi Key Laboratory of Environmental Exposomics and Entire Lifecycle Health, Guilin Medical University, Guilin, China; 2 Department of Environmental and Occupational Health, School of Public Health, Guilin Medical University, Guilin, China

**Keywords:** bisphenol S, ferroptosis, liver damage, mitochondrial damage, oxidative stress

There was a mistake in Figure 2H as published. Our results demonstrated that BPS reduced GSH levels, and ferroptosis inhibitors Fer-1 could reverse the BPS-induced decline in GSH. Unfortunately, Figure 2H was inconsistent with the corresponding results description. The corrected Figure 2H appears below.

**FIGURE 2:**
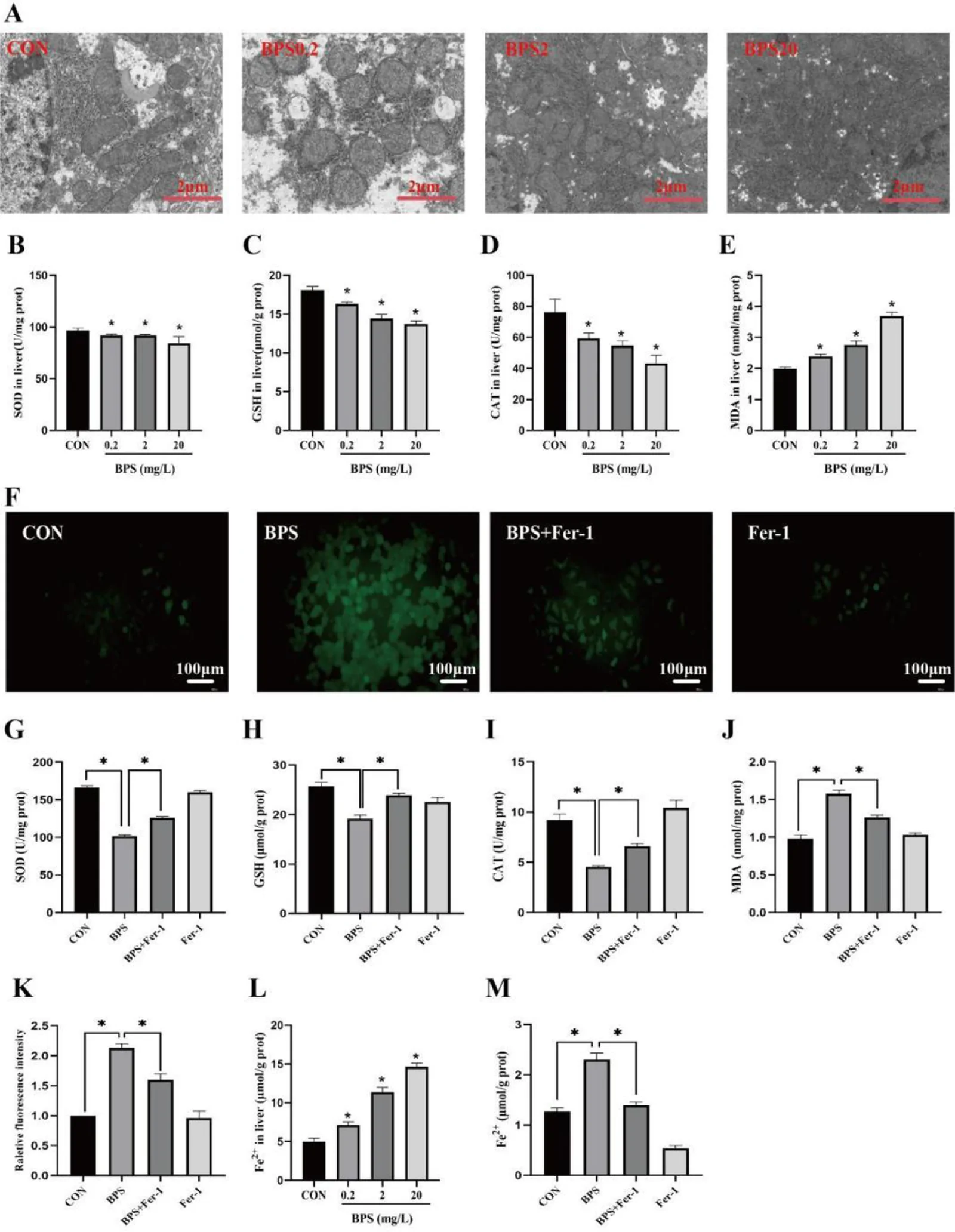
BPS induces ferroptosis in the liver. **(A)** The ultrastructure of liver mitochondria. Scale bars = 2 µm. **(B–E)** BPS changed the indicators of hepatic antioxidant of mouse live tissues (n = 6, *P < 0.05). **(F,K)** The fluorescence intensity of ROS in the AML12 cells (×200 magnification). **(G–J)** BPS changed the indicators of hepatic antioxidant of AML12 cells (n = 6, *P < 0.05). **(L)** The Fe^2+^ levels in the liver tissues (n = 6, *P < 0.05 vs. CON). **(M)** The Fe^2+^ levels in the AML12 cells (n = 6, *P < 0.05).

The original version of this article has been updated.

