# Supplementary material for: Pregnancy and lactation exposure to bisphenol S induces ferroptosis via disrupted hepatic lipid metabolism in offspring mice
Source: Front Toxicol. 2026 Jun 5;8:1753029. doi: 10.3389/ftox.2026.1753029 (PMC13278686; doi:10.3389/ftox.2026.1753029)
Supplement: Supplementary file 1 [file DataSheet1.docx]

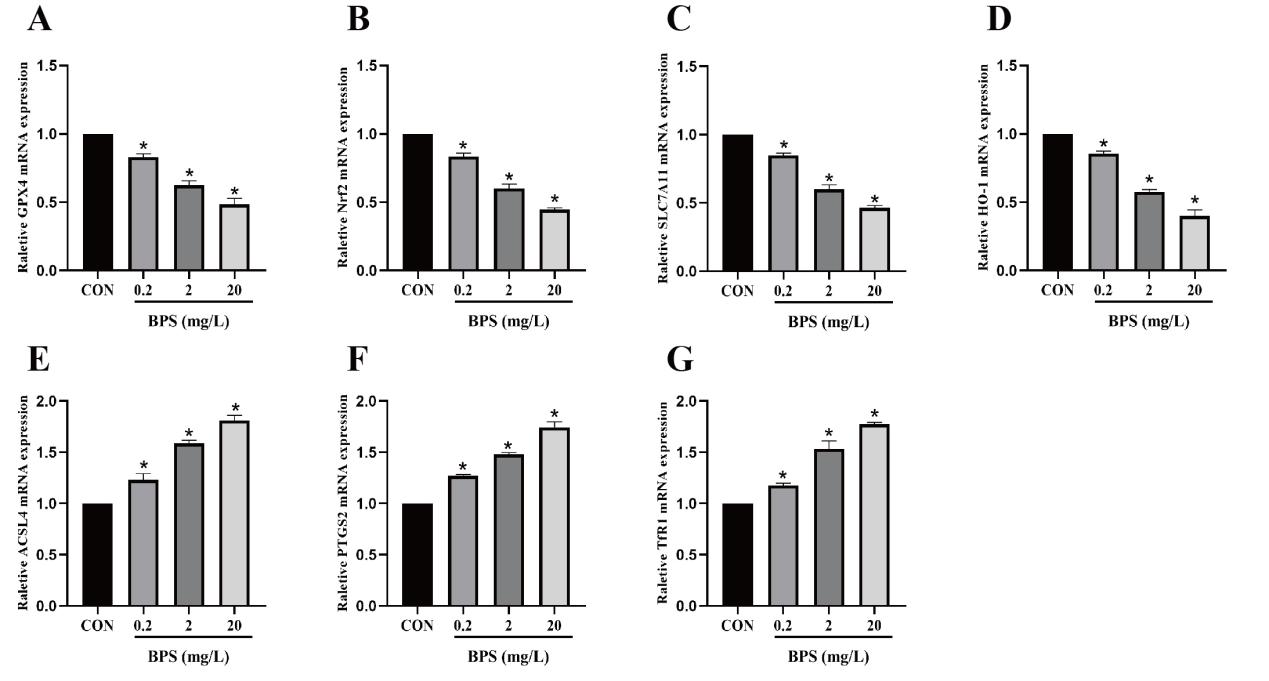


**Fig. S1.** The mRNA expression level of ferroptosis-related genes in mouse liver tissue. (A) *GPX4*; (B) *Nrf2*; (C) *SLC7A11*; (D) *HO-1*; (E) *ACSL4*; (F) *PTGS2*; (G) *TfR1*. (n =3, **P* < 0.05 vs. CON).


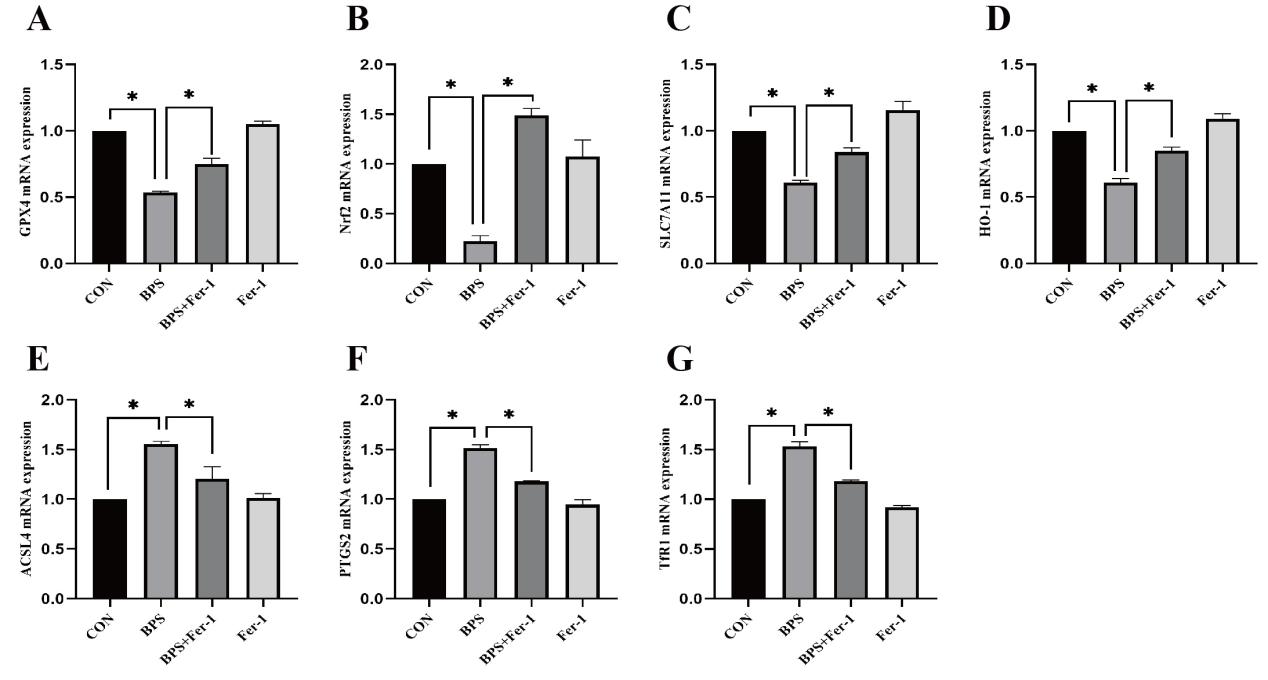


**Fig. S2.** The mRNA expression level of ferroptosis-related genes in AML12 cells. (A) *GPX4*; (B) *Nrf2*; (C) *SLC7A11*; (D) *HO-1*; (E) *ACSL4*; (F) *PTGS2*; (G) *TfR1*. (n =3, **P* < 0.05).


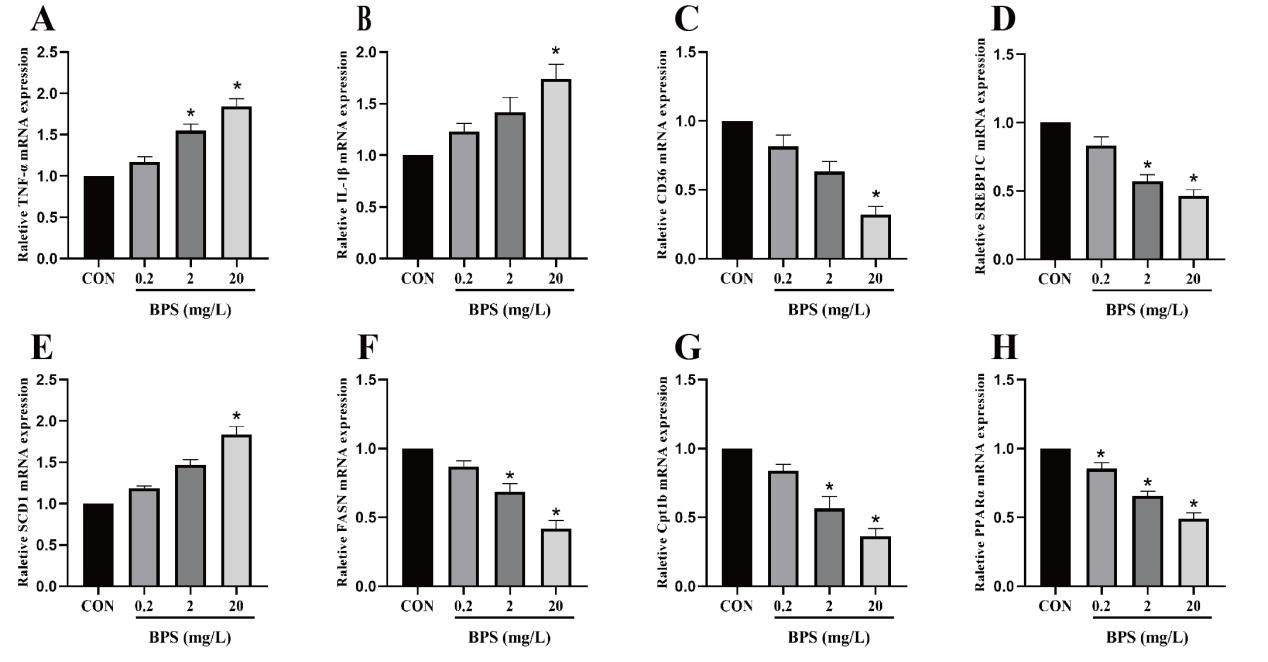


**Fig. S3.** The mRNA expression level of hepatic lipid metabolism-related genes. (A) *TNF-α*; (B) *IL-1β*; (C) *CD36*; (D) *SREBP1C*; (E) *SCD1*; (F) *FASN*; (G) *Cpt1b*; （H） *PPARα*. (n =3, **P* < 0.05 vs. CON).


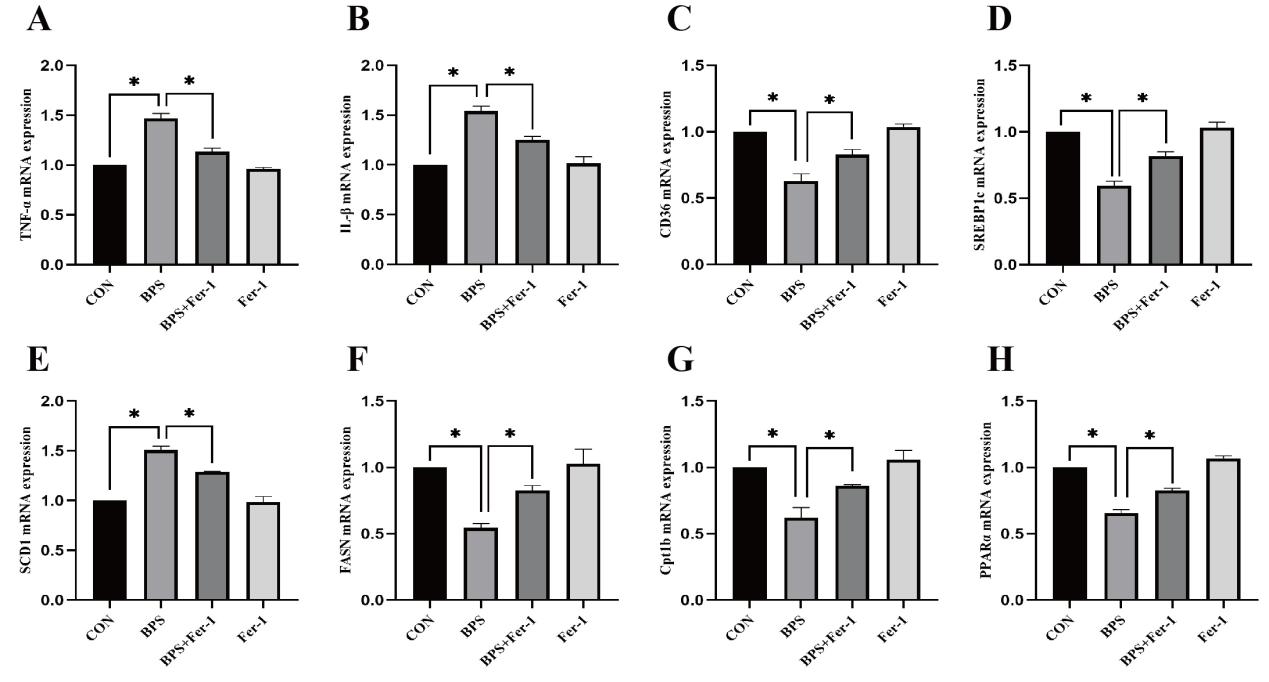


**Fig. S4.** The mRNA expression level of lipid metabolism-related genes in AML12 cell. (A) *TNF-α*; (B) *IL-1β*; (C) *CD36*; (D) *SREBP1C*; (E) *SCD1*; (F) *FASN*; (G) *Cpt1b*; （H） *PPARα*. (n =3, **P* < 0.05).
